# Area 55b forms a cortical transition zone for sensorimotor-semantic transformation in human language

**DOI:** 10.21203/rs.3.rs-9934100/v1

**Published:** 2026-06-08

**Authors:** Guowei Wu, Xiuyi Wang, Baihan Lv, Yi Du

**Affiliations:** 1. State Key Laboratory of Cognitive Science and Mental Health, Institute of Psychology, Chinese Academy of Sciences, Beijing, China.; 2. Department of Psychology, University of Chinese Academy of Sciences, Beijing, China.

**Keywords:** area 55b, language, sensorimotor–semantic interface

## Abstract

Human language requires seamless transformations between amodal concepts and modality-specific sensorimotor systems, yet the mediating cortical interface remains elusive. Using individualized functional mapping across word comprehension (listening and reading) and production (naming and writing) tasks, we evaluated the modality-invariant lexical network against five stringent, *a priori* criteria. We identified left area 55b as the exclusive sensorimotor–semantic interface. Area 55b robustly distinguished comprehension from production, exhibited production-enhanced semantic representations, and dynamically reconfigured its effective connectivity to motor-effector systems during production. Crucially, it revealed an internal anterior-posterior functional gradient: an anterior language-network component carried stronger semantic information, whereas a posterior somato-cognitive action-network component carried stronger articulatory information. This organization was supported by white matter connectivity and positioning along macroscale cortical hierarchies. Together, these findings reveal area 55b as a cortical transition zone for sensorimotor–semantic transformation, providing a mechanistic account of how language bidirectionally bridges meaning with perception and action.

## Introduction

A fundamental challenge in neuroscience is the "interface problem"—understanding how the brain bridges the divide between abstract, amodal representations and modality-specific physical actions or sensory inputs. Human language perfectly exemplifies this required flexibility: a single concept can be accessed through diverse sensory inputs and expressed through different motor outputs. This capacity necessitates a neural architecture that links amodal conceptual representations with modality-specific sensory and motor systems ^1-3^. Current models largely partition this architecture into two interacting domains: a conceptual system, anchored by regions like the anterior temporal lobe, which represents meaning independent of input or output modality ^4-6^, and language-relevant perceptual and motor systems that process the surface forms of linguistic signals—including speech sounds, visual word forms, and articulatory or writing codes—without directly engaging their inherent meaning ^1,7^. However, because the precise cortical interface linking these meaning-sensitive systems to surface-form-sensitive systems remains unresolved, existing mechanistic accounts of lexical processing are fundamentally fragmented. While current models successfully delineate isolated semantic ^8-10^, perceptual ^11-13^ and motor components ^14-16^, they fail to provide an explicit account of the cortical mechanism that executes the transformation required for fluid language use. Consequently, the field lacks a cohesive framework for identifying regions that do more than merely participate in lexical tasks, but instead support semantic–sensorimotor transformation.

Two pervasive obstacles—one conceptual, one spatial—have hindered progress in identifying this interface. Conceptually, the field has lacked a principled definition of what a genuine sensorimotor–semantic interface actually does. Existing accounts assume shared semantic representations across comprehension and production, as well as neural mechanisms linking sensorimotor processes to semantic representations ^1,17,18^. Yet, these accounts have not specified the unique functional signature of the interface itself. Without explicit functional criteria, candidate regions are often nominated based merely on cross-modal recruitment or lexical involvement, without establishing a true semantic-to-sensorimotor transformation. For instance, the middle fusiform/visual word form area (VWFA) supports perceptual–semantic convergence ^19,20^, but is primarily tied to visual–orthographic processing and does not account for bidirectional transformations required for both comprehension and production. Similarly, the Sylvian parietal-temporal area is largely restricted to auditory–motor integration rather than conceptual or semantic transformations ^21-23^. Thus, cross-modal recruitment alone is insufficient to establish interface status.

Spatially, traditional group-averaged brain parcellations can blur fine-grained areal boundaries and obscure functionally meaningful transitions ^24-27^. If a semantic–sensorimotor interface is localized, it is likely to lie near a fine-scale transition between semantic-facing and sensorimotor-facing systems, making it especially vulnerable to inter-individual variability ^28,29^. This is especially relevant for highly variable regions like area 55b, whose localization and surrounding transitions are frequently mischaracterized by coarse group averages ^30,31^. Resolving this problem therefore requires both explicit functional criteria for adjudicating interface status and individualized cortical mapping to preserve fine-grained functional organization.

We therefore operationally defined a sensorimotor–semantic interface using five a priori, location-agnostic criteria. A candidate interface region linking conceptual representations with modality-specific sensorimotor systems should: (1) distinguish comprehension from production across sensory and motor formats, as these task domains place different demands on the transformation between meaning and sensorimotor representations; (2) exhibit enhanced semantic representations during production, when conceptual content must be converted into output-specific sensorimotor codes ^32^; (3) reconfigure its effective connectivity between input-related and output-related systems across comprehension and production; (4) contain an internal functional organization separating semantic-facing from sensorimotor-facing representations, supported by dissociable white-matter connectivity; and (5) occupy a local transition zone along macroscale cortical sensory-to-association hierarchies.

**Fig. 1 ∣:**
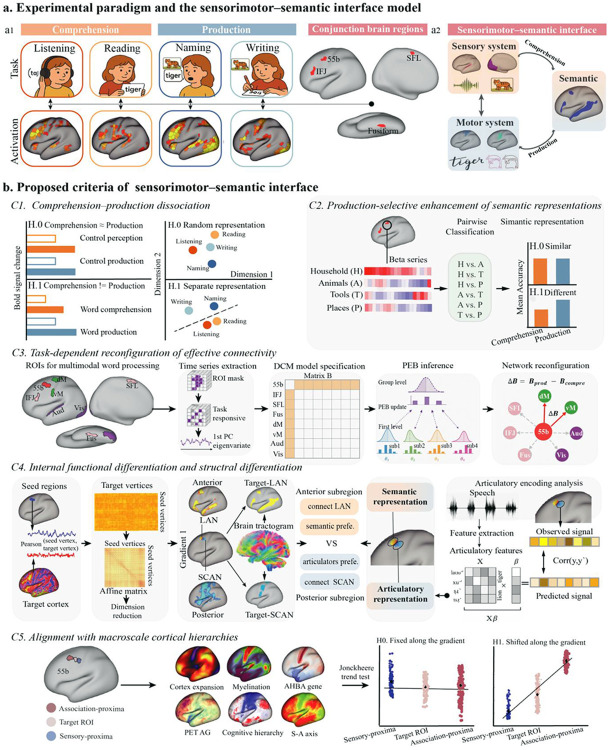
Experimental paradigm and hierarchical framework for identifying the sensorimotor–semantic interface. **a**, Experimental paradigm and the sensorimotor–semantic interface model. **a1**, Participants performed four lexical tasks spanning comprehension (listening, reading) and production (naming, writing), each contrasted against its modality-matched non-lexical control condition. A conjunction analysis across all four tasks identified a format-independent lexical network. **a2**, The theoretical model defines the sensorimotor–semantic interface as a site supporting bidirectional transformations between modality-specific sensory/motor representations and amodal semantic concepts during language comprehension and production. **b,** The five *a priori* criteria (C1–C5) and analytical workflow for identifying candidate interface regions. C1, Comprehension–production dissociation in both activation magnitude and multivariate patterns: an interface should show a production-biased profile, expressed as stronger or separable responses during production than comprehension. C2, Production-selective enhancement of semantic representations: semantic representations should be stronger during production than comprehension, assessed by comparing semantic decoding accuracy across tasks. C3, Task-dependent reconfiguration of effective connectivity: an interface region should reconfigure its directed interactions according to task direction, with production increasing coupling from the interface to motor-effector systems than comprehension. This was tested using a sparse area 55b-centered dynamic causal modelling framework that estimated task-dependent modulation of directed connections between area 55b and sensory, motor and lexical/associative nodes. C4, Internal functional and structural differentiation: an interface should contain internally differentiated subregions that preferentially support semantic-facing and sensorimotor-facing functions. This was tested by deriving subject-specific local functional gradients within area 55b and examining whether gradient-defined subregions differed in semantic decoding, articulatory-feature encoding and white-matter connectivity to language and action networks. C5, Alignment with macroscale cortical hierarchies: internal differentiation within the interface region should align with local transitions along established cortical hierarchies. This was tested by quantifying ordered transitions across multiple hierarchy maps and comparing their strength and consistency with those in control regions. Abbreviations: 55b, area 55b; AHBA, Allen Human Brain Atlas; Aud, auditory cortex; Compre, comprehension; dM, dorsal hand motor cortex; Fus, anterior fusiform gyrus; IFJ, inferior frontal junction; PC, principal component; PET AG, PET-derived aerobic glycolysis; prefe., preference; Prod, production; S-A axis, sensory-to-association hierarchy; SFL, superior frontal language area; Vis, visual cortex; vM, ventral laryngeal–orofacial motor cortex.

To test this framework, we combined four word-level tasks spanning comprehension (listening, reading) and production (naming, writing) with individualized functional parcellation and multi-level analyses aligned with the proposed interface criteria (Fig. 1a and Supplementary Fig. 1 and 2). This design addressed prior limitations in three ways. First, by comparing comprehension and production across different input and output formats within the same participants, we distinguished interface-related functions from modality-specific effects. Second, using individualized parcellation preserved the sharp functional transitions necessary to identify a localized interface. Third, by evaluating activation, representational content, effective connectivity, internal organization, and cortical hierarchy, we tested interface status explicitly rather than inferring it from task engagement alone. Using this approach, we identified area 55b as the only candidate region satisfying all five criteria, positioning it as a cortical transition zone for semantic–sensorimotor transformation in human language.

## Results

### Identification strategy for a sensorimotor–semantic interface

To localize a sensorimotor–semantic interface with high functional precision, we analyzed the multimodal lexical task functional magnetic resonance imaging (fMRI) data using individualized functional parcellation based on the Schaefer–Kong framework ^24^. We first identified a modality-invariant lexical network by performing a conjunction analysis across two comprehension tasks, word listening and reading, and two production tasks, word naming and writing, each contrasted with its modality-matched control condition. We then evaluated all regions in this conjunction-defined network against five *a priori*, location-agnostic criteria for a sensorimotor–semantic interface: (1) a dissociation between comprehension and production (C1); (2) selective enhancement of semantic representations during word production (C2); (3) task-dependent reconfiguration of effective connectivity between comprehension and production (C3); (4) an internal anterior-posterior organization separating semantic from articulatory representations and supported by dissociable white matter connectivity (C4); and (5) convergence at local transitions along multiple macroscale cortical hierarchies (C5). This workflow is summarized in Fig. 1. While several regions were recruited across modalities, only area 55b satisfied all five criteria, as detailed below.

### A modality-invariant lexical network includes area 55b, SFL, IFJ and anterior fusiform gyrus

We hypothesized that an interface region should be recruited across both sensory and motor modalities during word processing. A conjunction analysis across four task-specific contrasts — word listening and reading for comprehension, and word naming and writing for production — identified four regions showing significantly greater activation for lexical tasks than for their corresponding modality-matched control conditions: area 55b, the superior frontal language area (SFL; defined using the Glasser 360 atlas ^31^), the inferior frontal junction (IFJ), and the anterior fusiform gyrus (FDR-*q* < 0.05; Table 1 and Fig. 1a).

This pattern was replicated in a vertex-wise analysis (Supplementary Fig. 3), indicating that the finding was independent of parcel granularity. We further confirmed the robustness of this network in an independent validation dataset, the Peking University dataset ^19^. In this sample (n = 100), all four regions were recruited across the word comprehension and production tasks (Supplementary Results *Validation of candidate lexical-network activation in the Peking University Dataset* and Supplementary Fig. 4). Together, these analyses identified a robust modality-invariant lexical network comprising area 55b, SFL, IFJ, and anterior fusiform gyrus, which served as the candidate set for subsequent evaluation against the five interface criteria.

### Area 55b and SFL dissociate word comprehension from production tasks in activation and multivariate patterns (Criterion 1)

**Fig. 2 ∣:**
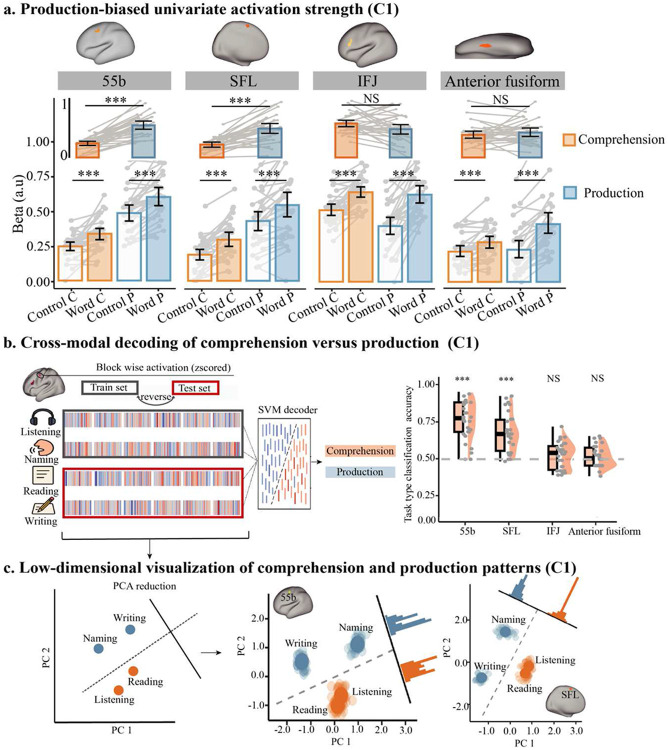
Area 55b and SFL dissociate word comprehension from production (C1). **a,** Production-biased univariate activation strength. While all four conjunction-defined regions (area 55b, superior frontal language area (SFL), inferior frontal junction (IFJ), and anterior fusiform gyrus showed significant lexical responses, only area 55b and SFL exhibited stronger activation during production than comprehension. Lower bars show mean activation magnitude for control (open) and word (filled) conditions; upper bars show the production–versus-comprehension contrast. Error bars denote SEM. **b,** Cross-modal decoding of comprehension versus production. Area 55b and SFL showed above-chance cross-modal classification of comprehension versus production, whereas IFJ and anterior fusiform gyrus did not. Box–violin plots show participant-level classification accuracy, the medians and interquartile ranges; the dashed line indicates chance level (0.50). **c,** Low-dimensional visualization of comprehension and production patterns. Consistent with the decoding results, comprehension (orange) and production (blue) patterns occupied separable positions in area 55b and SFL. Asterisks indicate Bonferroni-corrected significance (*** *P* < 0.001, ** *P* < 0.01, * *P* < 0.05); NS, not significant.

A genuine interface region must not only be engaged in lexical processing across modalities, but must also differentiate between comprehension and production tasks, as production places greater demands on mapping semantic content onto sensorimotor representations than comprehension. We first tested this prediction by comparing univariate activation magnitudes between production tasks (naming and writing) and comprehension tasks (listening and reading). Both area 55b and SFL showed a production-biased profile, with significantly stronger activation during production than comprehension tasks (area 55b: *t*(29) = 9.226, *P* < 0.001, Cohen’s *d* = 1.68; SFL: *t*(28) = 6.752, *P* < 0.001, Cohen’s *d* = 1.25; Fig. 2a). By contrast, the IFJ and anterior fusiform gyrus did not show a significant production–comprehension difference (IFJ: *t*(29) = −2.272, *P* = 0.153, Cohen’s *d* = −0.415; anterior fusiform gyrus: *t*(28) = 2.729, *P* = 0.054, Cohen’s *d* = 0.51). All *P* values were Bonferroni-corrected across the four candidate regions. This production-biased activation profile was replicated in the Peking University dataset (Supplementary Result: *Validation of production-based activation in the Peking University Dataset* and Supplementary Fig. 4).

We next asked whether activation patterns within these regions could distinguish comprehension from production independent of sensory input or motor output modality. To test this, we used cross-modal multivariate pattern analysis (MVPA): classifiers were trained on one comprehension–production pair (e.g., listening versus writing) and tested on another pair (e.g., reading versus naming). This design tested for comprehension–production information that generalized across modalities while reducing reliance on modality-specific sensory or motor features. Successful decoding therefore indicates the presence of modality-invariant information regarding task demands.

Consistent with the univariate results, area 55b and SFL showed significantly above-chance cross-modal classification of task type (area 55b: mean accuracy = 0.76, SD = 0.13; *t*(29) = 10.74, *P* < 0.001, Cohen’s *d* = 1.96; SFL: mean accuracy = 0.67, SD = 0.13; *t*(29) = 7.494, *P* < 0.001, Cohen’s *d* = 1.37). Notably, classification accuracy was significantly higher in area 55b than in SFL (*t*(29) = 3.05, *P* = 0.005, Cohen’s *d* = 0.56; Fig. 2b). Conversely, neither the IFJ nor anterior fusiform gyrus showed above-chance classification (IFJ: mean accuracy = 0.50, SD = 0.09; *t*(29) = 0.28, *P* = 1, Cohen’s *d* = 0.05; anterior fusiform gyrus: mean accuracy = 0.52, SD = 0.07; *t*(29) = 1.28, *P* = 0.42, Cohen’s *d* = 0.23).

To intuitively visualize the structure of these multivariate patterns, we projected task-specific activation patterns into a low-dimensional space using principal component analysis (PCA; Fig. 2c). Consistent with the classification results, comprehension and production patterns occupied separable positions in area 55b and SFL. Crucially, this dissociation could not be explained by orthographic task grouping: using the same cross-modal framework, neither area 55b nor SFL could decode orthographic tasks (reading and writing) from non-orthographic tasks (listening and naming) at above-chance levels (area 55b: *P* = 1.00, Cohen’s *d* = −0.12; SFL: *P* = 1.00, Cohen’s *d* = −0.09; Supplementary Results: *Orthographic-form classification does not explain comprehension–production decoding in area 55b and SFL*). Together, these results indicate that only area 55b and SFL showed both a production-biased activation profile and modality-invariant multivariate information that differentiates comprehension from production. Accordingly, these two regions were carried forward for subsequent interface tests.

### Semantic representations are selectively enhanced in area 55b during word production (Criterion 2)

Having established that area 55b and SFL distinguished comprehension from production across modalities, we next tested a key mechanistic prediction: an interface region must support the transformation of conceptual information into sensorimotor output codes, and should therefore show stronger semantic representations during production than during comprehension. To test this, we decoded semantic category information from multivariate response patterns in area 55b and SFL using pairwise classification among four categories and compared decoding accuracy between production and comprehension tasks.

**Fig. 3 ∣:**
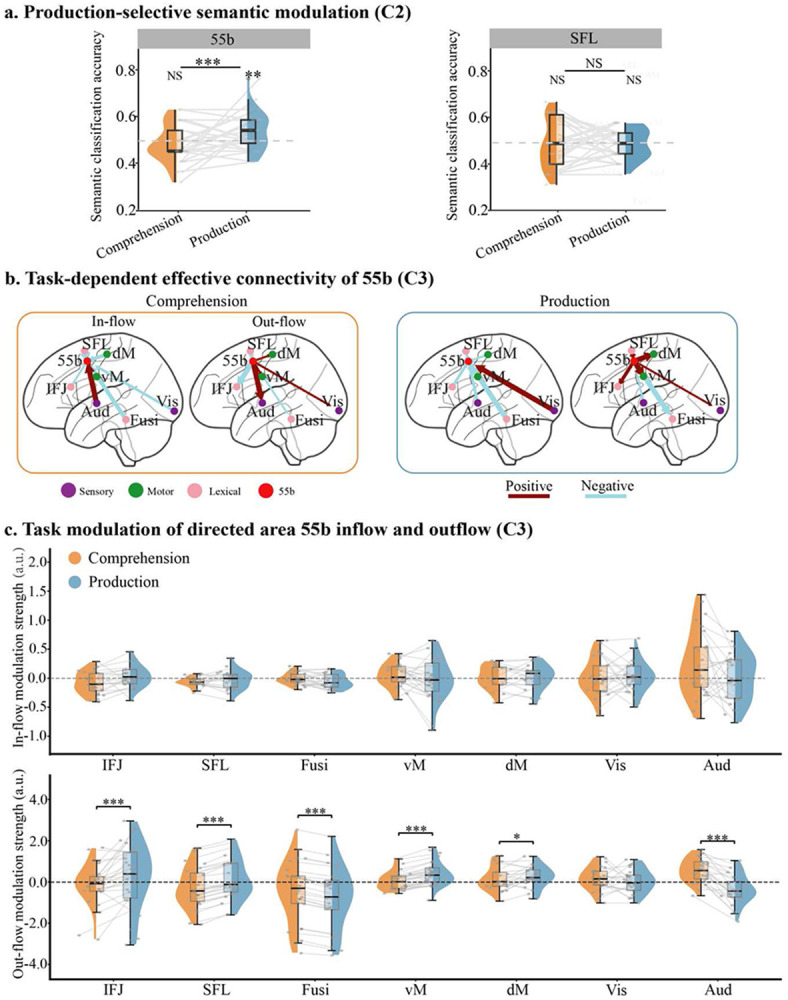
Area 55b shows production-selective semantic decoding (C2) and reweights output toward frontal–motor systems during production (C3). **a,** Production-selective semantic modulation (C2). Area 55b showed significantly greater semantic category classification accuracy during production than comprehension, with above chance decoding only during production. SFL showed no production–comprehension difference, and decoding did not exceed chance in either task domain. The dashed line indicates chance level (0.50). Orange denotes comprehension and blue denotes production; grey lines connect paired participant estimates. **b**, Task-dependent effective connectivity of area 55b (C3). A sparse area 55b-centred DCM estimated bidirectional connections between area 55b and input-related, lexical and effector-specific motor regions. Inflow to and outflow from area 55b are shown separately for comprehension and production. Arrow direction indicates directed influence; line thickness scales with the absolute group-mean modulation parameter; red and light blue denote positive and negative modulation, respectively. **c**, Production–comprehension differences in directed inflow and outflow involving area 55b (C3). Inflow connections showed no significant task-domain differences after FDR correction, whereas outflow connections were selectively reweighted during production toward frontal lexical and effector-specific motor regions. Box plots show medians and interquartile ranges; violin widths indicate data density; grey lines connect paired participant estimates after outlier exclusion. Asterisks denote FDR-corrected significance (*q < 0.05, **q < 0.01, ***q < 0.001); NS, not significant. Abbreviations: Aud, auditory cortex; Vis, visual cortex; Fus, anterior fusiform gyrus; SFL, superior frontal language area; IFJ, inferior frontal junction; vM, ventral laryngeal–orofacial motor cortex; dM, dorsal hand motor cortex.

Area 55b, but not SFL, demonstrated a selective enhancement of semantic representations during production (Fig. 3a). In area 55b, semantic decoding accuracy was significantly higher during production than comprehension (Δaccuracy = 0.06; *t*(28) = 2.74, FDR-*q* = 0.045, Cohen’s *d* = 0.54). Decoding accuracy in area 55b significantly exceeded chance during production (chance = 0.50; mean accuracy = 0.54, SD = 0.08; FDR-*q* = 0.023, Cohen’s *d* = 0.48), but not during comprehension (chance = 0.50, mean accuracy = 0.49, SD = 0.08; FDR-*q* = 0.82, Cohen’s *d* = −0.18). In contrast, SFL showed no production–comprehension difference in decoding accuracy (*t*(29) = 0.28, FDR-*q* = 0.87, Cohen’s *d* = −0.05), and decoding accuracy did not exceed chance during either production (mean accuracy = 0.50, SD = 0.06, FDR-*q* = 0.685) or comprehension (mean accuracy = 0.50, SD = 0.10, FDR-*q* = 0.685). Together, these results indicate that, among the two regions carried forward from Criterion 1, only area 55b exhibited production-selective semantic decoding. Therefore, area 55b was prioritized as the primary candidate interface locus for subsequent criterion-based tests.

### Area 55b reconfigures its effective connectivity toward motor-effector systems during production (Criterion 3)

**Figure 4 ∣:**
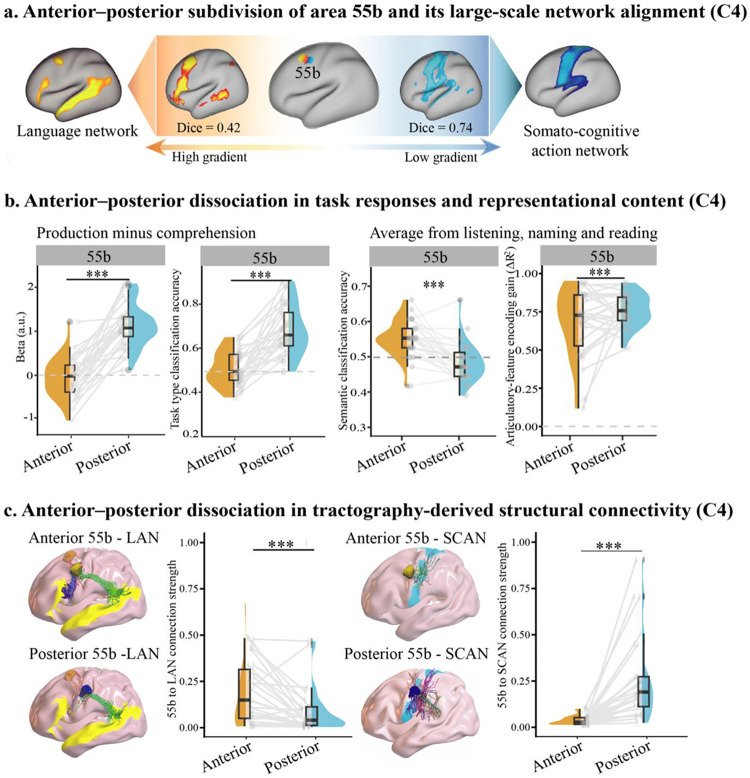
Area 55b shows a structurally supported anterior–posterior organization separating semantic and articulatory representations. **a,** Anterior–posterior subdivision of area 55b and its large-scale network alignment (C4). Based on local connectivity gradients, area 55b was partitioned into an anterior high-gradient subregion (warm color), which aligned most strongly with the language (LAN) network, and a posterior low-gradient subregion (cold color), which aligned most strongly with the somato-cognitive action network (SCAN). Dice coefficients indicate spatial overlap between each 55b subregion and its best-matching large-scale network. **b,** Anterior–posterior dissociation in task responses and representational content within area 55b. Compared to the anterior subregion, the posterior subregion showed stronger production-biased activation contrast defined as production minus comprehension(left) and higher classification accuracy for distinguishing comprehension from production (center-left). Furthermore, the anterior subregion showed greater semantic category decoding accuracy than the posterior subregion (center-right), whereas the posterior subregion exhibited stronger articulatory-feature encoding than the anterior subregion (right). Articulatory-feature encoding gain denotes the improvement of the articulatory-feature model over the baseline model. Dashed lines indicate the null value for activation contrasts and chance level for classification analyses. **c,** Anterior–posterior dissociation in tractography-derived structural connectivity (C4). The anterior subregion showed stronger structural connectivity with the language network (LAN) than the posterior subregion, whereas the posterior subregion showed stronger structural connectivity with SCAN than the anterior subregion. This crossed structural connectivity profile paralleled the anterior semantic/language-facing and posterior action/articulatory-facing organization observed in functional analyses. Tract renderings from an example participant illustrate streamlines connecting anterior and posterior 55b seeds with LAN and SCAN targets. For box–violin plots, boxes indicate medians and interquartile ranges, violin widths indicate data density and grey lines connect paired participant estimates across subregions. Asterisks denote FDR-corrected significance (*q < 0.05, **q < 0.01, ***q < 0.001).

A sensorimotor–semantic interface should reconfigure its effective connectivity according to the direction of language processing. We therefore tested whether directed coupling between area 55b and task-relevant input, lexical and output regions differed between comprehension and production, with the key prediction that production would selectively increase directed information flow from area 55b to production-related lexical and motor-effector regions. We estimated effective connectivity within an eight-node network selected to cover the input, lexical and output components of the four tasks: auditory, visual input-related regions; modality-general lexical regions including area 55b, SFL IFJ, and fusiform gyrus; and laryngeal–orofacial and hand motor regions supporting spoken naming and writing. We used a sparse, area 55b-centred dynamic causal modelling (DCM) in which bidirectional connections were estimated between area 55b and each of the other seven nodes, while non-55b connections were not modelled (Fig. 4b; see Methods, Testing C3: Task-dependent reconfiguration of effective connectivity via DCM, for ROI definitions and model specification). This model tested task-dependent changes in directed coupling along connections involving the candidate interface region.

Production selectively increased outgoing effective connectivity from area 55b to motor-effector and frontal lexical/language regions (Fig. 4c). Relative to comprehension, production increased directed coupling from area 55b to both effector-specific motor regions: the ventral laryngeal–orofacial motor region involved in spoken naming (mean difference, production – comprehension = 0.324; *t*(29) = 11.40, FDR-*q* < 0.001, Cohen’s *d* = 2.08) and the dorsal hand motor region involved in writing (mean difference = 0.302; *t*(25) = 3.63, FDR-*q* = 0.002, Cohen’s *d* = 0.71). Similar production-related increases were observed in two frontal regions within the modality-invariant lexical network: IFJ (mean difference = 0.746; *t*(29) = 5.00, FDR-*q* < 0.001, Cohen’s *d* = 0.91) and SFL (mean difference = 0.413; *t*(29) = 15.97, FDR-*q* < 0.001, Cohen’s *d* = 2.92). Thus, during conceptual-to-output transformation, area 55b showed stronger directed influence on both motor implementation systems and frontal lexical/language regions.

We next tested whether this production-related increase reflected selective output reweighting rather than a global increase in all outgoing influences from area 55b. The 55b-to-visual cortex connection did not differ between production and comprehension (mean difference = −0.165; *t*(26) = −1.04, FDR-*q* = 0.309, Cohen’s *d* = −0.20). By contrast, two pathways showed the opposite pattern: 55b-to-auditory cortex modulation was stronger during comprehension than production, as reflected by a significant production-related decrease (mean difference = −0.909; *t*(27) = −5.79, FDR-*q* < 0.001, Cohen’s *d* = −1.09), and 55b-to-fusiform modulation was also reduced during production relative to comprehension (mean difference = −0.308; *t*(28) = −21.49, FDR-*q* < 0.001, Cohen’s *d* = −3.99). Thus, the production-related increase in 55b output was selectively directed toward frontal motor-effector and language-related systems.

This task-dependent reconfiguration was expressed primarily in outgoing rather than incoming connectivity. None of the seven incoming connections to area 55b showed a reliable production–comprehension difference after FDR correction (Fig. 4c, Supplementary Table 1). Thus, the effective-connectivity profile was not characterized by a broad change in afferent influence to area 55b, but by selective reweighting of its outgoing directed influences.

Together, these results show that area 55b reconfigured its directed interactions according to task direction, preferentially increasing outgoing coupling to frontal language-related and motor-effector systems during production. This task-dependent routing profile supports the role of area 55b as a sensorimotor–semantic interface.

### Area 55b shows a structurally supported anterior–posterior organization separating semantic and articulatory representations (Criterion 4)

Having identified area 55b as the primary interface candidate, we next asked whether its internal organization reflected a semantic-to-sensorimotor transition. If area 55b serves as a sensorimotor–semantic interface, its semantic-facing sector should be more closely coupled to the language network, represent semantic information more strongly, and show weaker bias toward production, whereas its sensorimotor-facing sector should be more closely coupled to sensorimotor/action systems, encode articulatory features more strongly, and exhibit stronger bias toward production. This division should also be supported by dissociable white-matter connectivity to language and sensorimotor/action networks.

To test this hypothesis, we derived a local connectivity gradient within area 55b and divided the region into anterior high-gradient and posterior low-gradient subregions (Fig. 4a). These subregions exhibited distinct large-scale network affiliations: the anterior subregion aligned most strongly with the canonical language network (Dice = 0.42), indicating integration with high-level linguistic processing, whereas the posterior subregion aligned most strongly with the somato-cognitive action network (SCAN; Dice = 0.74), signifying integration with sensorimotor/action systems. This anterior-posterior organization was replicated in both the HCP and Peking University datasets (Supplementary Fig. 6).

This anterior–posterior segregation strictly governed both univariate activation and multivariate task responses. The posterior subregion exhibited a significantly larger production–comprehension difference in univariate activation than the anterior subregion (*t*(29) = 10.01, FDR-*q* < 0.001, Cohen’s *d* = 1.83), and a stronger discrimination between comprehension and production in multivariate classification (*t*(29) = −3.64, FDR-*q* = 0.005, Cohen’s *d* = −0.67). When analyzed independently, the anterior subregion showed no significant difference in activation magnitude between production and comprehension (Meanβ (production - comprehension) = 0.01; *t*(29) = 0.20, FDR-*q* = 0.84; Fig. 4b, left) and did not distinguish the two task domains above chance in multivariate classification (mean accuracy = 0.52, SD = 0.09; *t*(29) = 0.92, FDR-*q* = 0.46; chance = 0.50). The posterior subregion, however, strongly preferred production over comprehension (Meanβ = 0.94; *t*(29) = 10.24, FDR-*q* < 0.001) and distinguished the task domains with high accuracy (mean accuracy = 0.72, SD = 0.13; *t*(29) = 8.93, FDR-*q* < 0.001). Crucially, this anterior–posterior division also extended to representational content. The anterior subregion showed greater semantic category decoding accuracy than the posterior subregion (anterior: mean = 0.53, SD = 0.11; posterior: mean = 0.48, SD = 0.10; *t*(29) = 2.74, FDR-*q* = 0.007), whereas the posterior subregion exhibited far greater articulatory-feature encoding than the anterior subregion (posterior: mean = 0.76, SD = 0.11; anterior: mean = 0.59, SD = 0.37; *t*(29) = −2.60, FDR-*q* = 0.007; Fig. 4b, right). Together, these findings indicate that area 55b is architecturally organized precisely as a sensorimotor–semantic interface, with its anterior end preferentially supporting semantic representation and its posterior end preferentially supporting production-related and articulatory processing.

To test whether this functional division was supported by structural connectivity, we compared tractography-derived connection strength from the anterior and posterior 55b subregions to the language network and SCAN. A 2 × 2 repeated-measures ANOVA revealed a significant network × subregion interaction (*F*(1,25) = 30.59, *P* < 0.001, generalized η^2^ = 0.223; Fig. 4c), indicating that the anterior–posterior difference in structural connectivity depended on the target network. The simple-effects analyses confirmed the predicted crossed pattern. The anterior 55b subregion was more strongly connected to the language network than the posterior subregion (anterior – posterior = 0.013, *t*(25) = 3.35, *P* = 0.003, Cohen’s *d* = 0.66). Conversely, the posterior 55b subregion was more strongly connected to SCAN than the anterior subregion (anterior – posterior = −0.283, *t*(25) = −5.35, P < 0.001, Cohen’s *d* = −1.05). Together, this crossed structural connectivity profile mirrors the representational division within area 55b, linking its anterior sector preferentially to language/semantic systems and its posterior sector preferentially to action/articulatory systems.

### Functional segregation of area 55b aligns with independent macroscale cortical hierarchies (Criterion 5)

**Fig. 5 ∣:**
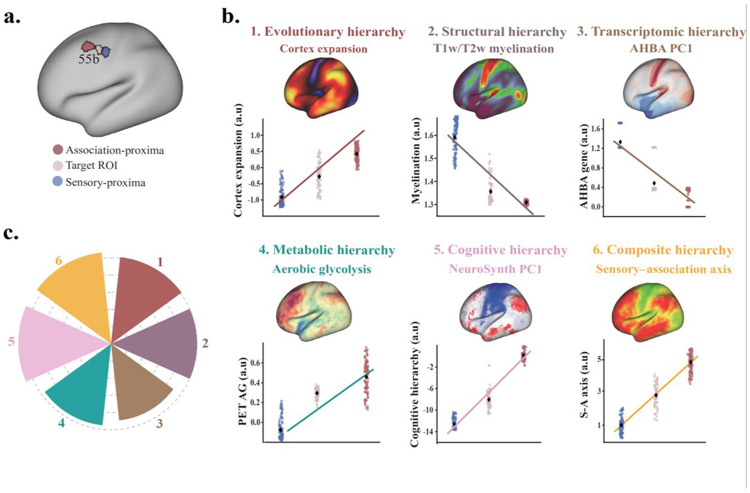
The functional segregation of area 55b aligns with macroscale cortical hierarchies (C5). **a,** Spatial sampling along the local sensory-to-association axis, capturing sensory-adjacent cortex (blue), the parcel containing area 55b (beige), and association-adjacent cortex (red). **b,** Multidimensional index of local hierarchical transition strength. The radial plot summarizes the strength of the monotonic transition across all six hierarchy maps; longer sectors indicate stronger transitions within area 55b. **c,** Consistent transitions across individual cortical hierarchies. Parcel values changed monotonically from sensory-adjacent cortex through the area 55b parcel to association-adjacent cortex across all six maps — an evolutionary hierarchy indexed by macaque-to-human cortical expansion, a structural/myeloarchitectonic hierarchy indexed by the T1w/T2w ratio, a transcriptomic hierarchy indexed by AHBA PC1, a metabolic hierarchy indexed by PET-derived aerobic glycolysis, a meta-analytic cognitive hierarchy indexed by NeuroSynth PC1, and a composite sensory-to-association hierarchy.

Finally, we asked whether the functional segregation within area 55b was embedded in broader principles of cortical organization. Because cortical hierarchies fundamentally structure the transformation from concrete sensorimotor representations to abstract transmodal states ^33^, we hypothesized that area 55b’s internal division should biologically coincide with a local transition from sensory-adjacent to association-adjacent cortex. To test this, we analyzed parcel values of three adjacent parcels spanning sensory-adjacent cortex, the parcel containing area 55b, and association-adjacent cortex across six independent macroscale cortical hierarchy maps (Fig. 5a). These maps captured complementary biological and functional dimensions of the sensory-to-association continuum: an evolutionary hierarchy indexed by macaque-to-human cortical expansion, a structural/myeloarchitectonic hierarchy indexed by the T1w/T2w ratio, a transcriptomic hierarchy indexed by AHBA PC1, a metabolic hierarchy indexed by PET-derived aerobic glycolysis, a meta-analytic cognitive hierarchy indexed by NeuroSynth PC1, and a composite sensory-to-association hierarchy (Fig. 5c; see Methods‘ section: Testing C5: Embedding of local functional segregation within established cortical hierarchies for detailed information).

Across all six maps, the transition spanning the sensory-adjacent parcel, through area 55b, and into the association-adjacent parcel followed a consistent, monotonic progression (all six ordered-trend tests *P* < 0.0001; Fig. 5b, 5c). This pattern places area 55b at a multidimensional cortical transition zone. Notably, this multidimensional hierarchical transition was significantly stronger and more consistent in area 55b than in any of the conjunction-defined or canonical language-network control regions (Supplementary Fig.7; Supplementary Table 2). Together, the five criteria converge on area 55b as a cortical transition zone for semantic–sensorimotor transformation.

## Discussion

A central challenge in language neurobiology is to characterize the mechanistic bridge between amodal conceptual knowledge and the modality-specific systems. Utilizing a multimodal word-processing paradigm spanning listening, reading, naming and writing, we provide convergent evidence that left area 55b serves as the dedicated sensorimotor–semantic interface for lexical processing. Although several regions are recruited across lexical tasks, only area 55b fulfills the stringent functional and architectural criteria of a genuine interface. It distinguishes comprehension from production across modalities, prioritizes semantic representations during conceptual-to-motor transformations, reconfigures its effective connectivity according to task direction, and exhibits an internal anterior–posterior organization separating semantic-facing and action-facing components. This internal organization was further supported by dissociable white-matter connectivity, with anterior 55b preferentially connected to the language network and posterior 55b preferentially connected to SCAN. Moreover, the anterior–posterior axis of area 55b is anchored within macroscale cortical hierarchies at the transition between sensorimotor and heteromodal association cortices. Together, these findings position area 55b as a cortical transition zone where lexical meaning is linked to the sensorimotor demands of language use.

Our findings help reconcile historically divergent accounts of area 55b by reframing it as an integrative interface rather than as a purely motor or purely semantic module. In classical dual-stream and dorsal-route models, the dorsal frontal speech regions have been characterized as part of a sound-to-articulation pathway, mapping phonological or acoustic speech representations onto articulatory plans ^11,12,34,35^. Consistent with this view, recent functional and structural studies have linked area 55b and adjacent posterior frontal language cortex to speech planning, syllabic sequencing and downstream articulatory control ^15,36^. Converging lesion and resection evidence also implicates the posterior middle frontal gyrus and putative area 55b in articulatory planning and semantic cognition ^37-39^. However, a purely motor-oriented account does not readily explain why this territory lies at the intersection of dorsal and ventral language pathways, connects with broad semantic networks, and encodes context-dependent semantic information even in the absence of overt articulation ^40-44^. Our results help resolve this apparent tension by showing that area 55b is internally differentiated along an anterior–posterior axis. The anterior component was more strongly aligned with semantic representations and the language network, whereas the posterior component was more strongly aligned with articulatory features and action-related systems. This organization provides a cortical architecture through which semantic information and motor implementation demands can be locally coordinated.

The interface role of area 55b was further supported by its task-dependent effective connectivity. Relative to comprehension, production selectively strengthened outgoing effective modulation from area 55b to motor-effector regions, including ventral laryngeal–orofacial and dorsal hand motor cortices. Production also enhanced coupling from area 55b to frontal lexical/language regions, including IFJ and SFL, suggesting increased interaction with frontal systems during conceptual-to-motor transformation. Importantly, this production-related increase was not a global amplification of all outgoing influences from area 55b; auditory and fusiform pathways showed stronger modulation during comprehension. This task-dependent routing profile extends emerging evidence that speech perception and production are mediated by state-specific connectivity shifts in dorsal frontal language regions ^45^, and suggests that area 55b flexibly couples lexical representations to the systems most relevant for the current direction of language processing.

The interface role of area 55b was evident in its internal organization. The anterior component more closely aligned with the language network and the posterior component more closely aligned with the SCAN network. This internal architecture refines prior proposals that the posterior frontal/area 55b territory acts as a broad integrative hub within the language system, bridging dorsal and ventral pathways and participating in semantic processing ^40,41^. Furthermore, it directly links the posterior subregion of area 55b to recent evidence that SCAN-adjacent motor-association territories support abstract, coordinated action planning rather than simple effector-specific outputs ^46,47^.

The significance of these findings extends beyond the language network, demonstrating how complex human cognitive abilities exploit fundamental principles of whole-brain organization. The anterior–posterior organization within area 55b was not an isolated topographical feature; it systematically aligned with local transitions across six independent, macroscale cortical hierarchy maps, tracing a path from sensory-adjacent to association-adjacent cortex. Because cortical hierarchies are thought to fundamentally structure the continuous transformation from concrete sensorimotor representations to abstract, transmodal representations ^2,3,48,49^, this position places area 55b at a highly biologically plausible watershed boundary for linking conceptual abstractions with concrete implementation codes.

This mechanistic framework finally clarifies the specific taxonomy of hubs within the broader lexical system. Semantic hub regions, particularly the anterior temporal lobe, are optimized to represent conceptual knowledge in an entirely amodal format ^4-6^. Conversely, ventral occipitotemporal regions support visual-form processing and perceptual–semantic convergence ^19,20^, while dorsal auditory–motor circuits specialize in phonological–articulatory mapping^12,34,50^. While these systems successfully explain isolated components of word processing, they fail to explain how lexical meaning is reformatted into the modality-specific codes required to actively use language (speaking, writing, reading and listening). Area 55b occupies this missing level of computational description: a local interface between conceptual representation and sensorimotor implementation.

A critical next step for the field will be establishing the causal necessity of this localized transformation zone. While individualized mapping and cascaded functional analyses strongly identify area 55b as the primary interface, they do not inherently prove that disrupting this region selectively impairs the mapping between lexical meaning and modality-specific sensorimotor codes. Future research using individualized stimulation and precision lesion-symptom mapping in aphasia could directly test whether perturbation or damage to area 55b selectively affects the conversion of meaning into spoken or written output, or the linking of sensory word forms to meaning, while preserving basic sensory and amodal semantic processing. Additionally, identifying area 55b as an interface does not imply that this transformation is implemented by a single cortical node. Understanding how area 55b interacts with sensorimotor, language and domain-general executive networks to orchestrate these transitions will be essential for mapping the full temporal dynamics of language use.

In conclusion, our findings identify left area 55b as a sensorimotor–semantic interface that links lexical meaning to the modality-specific demands of language use. Rather than functioning as a monolithic semantic, phonological or motor region, area 55b occupies a transitional position along the macroscale cortical hierarchy to actively bridge language-aligned and action-aligned neural systems and flexibly reconfigures its interactions during comprehension and production. By detailing how stable semantic abstractions are transformed into dynamic sensorimotor actions, this work provides a unified cortical account of the extraordinary flexibility that defines human language.

## Methods

### Datasets overview

This study used three neuroimaging datasets: a multimodal word-processing dataset collected by our team at the Institute of Psychology, Chinese Academy of Sciences (CAS); a published multimodal word dataset from Peking University ^19^; and a public resting-state fMRI dataset from the Human Connectome Project (HCP) ^51^. The CAS multimodal word-processing dataset served as the primary dataset for analyses. The Peking University dataset ^19^ was used to assess the robustness and cross-dataset generalizability of our findings. Resting-state fMRI data in the HCP S900 release were used to characterize local functional connectivity gradients.

### CAS multimodal word-processing dataset

#### Participants

Thirty-three healthy adults took part in this experiment. Three participants did not complete all experimental sessions and were excluded, leaving a final sample of thirty participants (16 women; mean age + SD, 22.67 + 2.98 years; age range, 18–34 years). All participants were right-handed native Chinese speakers with normal hearing and normal or corrected-to-normal vision. All participants provided written informed consent before participation. This study was approved by the Ethics Committee of the Institute of Psychology, Chinese Academy of Sciences, and was conducted in accordance with the Declaration of Helsinki. Participants received monetary compensation after completing the study.

#### Tasks and procedures

The CAS multimodal word-processing dataset comprised four word-level language processing tasks, including two comprehension tasks (listening and reading) and two production tasks (naming and writing). Each task included an experimental condition designed to probe lexical processing in a specific input or output modality, together with a modality-matched control condition that preserved basic sensory or motor demands while minimizing lexical and semantic content, thereby providing a baseline for isolating lexical-semantic processing. All tasks were implemented in a block design with experimental and matched control blocks. To improve the reliability of within-subject activation estimates (see Supplementary Methods, Results and Fig. 2), each participant completed three scanning sessions separated by at least three days, ensuring independent sampling of task-related responses. Each session comprised four runs, one for each task. The procedure and timing of each task are shown in Supplementary Fig. 1.

All four word-level tasks used the same set of 41 disyllabic Chinese words. 16 words were grouped into 4 semantic categories — household appliances, places, mammals and vehicles — with 4 words in each category; each category formed one semantic block. The remaining 25 words were grouped into 5 phonological sets according to the onset syllable of the first character (shu, shi, xiang, luo and jing), with 5 words in each set; each set formed one phonological block. Each task included 4 semantic blocks, 5 phonological blocks and 6 modality-matched control blocks. Each semantic block contained 4 items and each phonological block contained 5 items. Among the 6 control blocks, 3 blocks each contained 4 items to match the semantic blocks, and the other 3 blocks each contained 5 items to match the phonological blocks. The full set of stimuli was presented in Supplementary Table 3. We counted word frequency, first-syllable frequency, character frequency and stroke count. For the production tasks, we selected object pictures corresponding to the target words from standardized picture databases with high naming agreement ^52,53^.

#### Listening task

In the listening task, participants listened to spoken words in the experimental blocks and reported at the end of each block whether any animal-related words had appeared. In the control blocks, they listened to acoustically matched reversed-speech stimuli and reported whether they had heard any meaningful speech ^19,20^. The spoken-word stimuli were generated using female voice text-to-speech synthesis and the control stimuli were created by applying full time reversal to each audio file, producing unintelligible stimuli that preserved low-level acoustic properties. Each block began with a 200-ms fixation cross and was followed by a semantic, phonological or control block. Within each block, each item was presented for 2,000 ms with a fixed 250 ms interstimulus interval. A probe question was presented for 2,500 ms at the end of each block. After each block, a probe question appeared for 2,500 ms. Blocks were separated by fixation intervals of 9–12 s (mean, 11 s), yielding a total run duration of 5 min 58 s.

#### Reading task

In the reading task, participants viewed Chinese disyllabic words during the experimental blocks and judged at the end of each block whether any animal terms had appeared. During the control blocks, they viewed Korean Hangul strings and judged whether any Chinese characters had appeared. Korean Hangul strings were used as a visual control to preserve low-level orthographic input while minimizing access to lexical-semantic content ^54,55^. Before the experiment, we confirmed that participants could not read or identify Hangul characters. Each block began with a 200-ms fixation cross. Stimuli were presented for 2,000 ms with a fixed 250-ms interstimulus interval, followed by a 2,500-ms response period at the end of each block. Blocks were separated by fixation intervals of 9–12 s, yielding a total run duration of 5 min 58 s.

#### Naming task

In the naming task, participants viewed nameable object pictures and named each object aloud during the experimental blocks, whereas during the control blocks they viewed scrambled versions of the same pictures and produced a fixed utterance (“1, 2″). Spoken responses were recorded during scanning using an external microphone. Before scanning, participants completed a naming practice session to ensure consistent labels for the object pictures. The scrambled images were created by randomly rotating pixel blocks to preserve low-level visual properties, such as luminance and contrast, while disrupting recognizable structure ^20^. Each block began with a 200-ms fixation cross, after which each stimulus was presented for 2,500 ms with a fixed 250-ms interstimulus interval. Blocks were separated by fixation intervals of 9–12 s, yielding a total run duration of 5 min 55 s.

#### Writing Task

In the writing task, participants viewed object pictures and wrote the corresponding Chinese disyllabic words in the experimental blocks and viewed geometric line drawings adapted from previous studies ^56,57^ and copied the figures in the control blocks. Responses were recorded using a custom fMRI-compatible tablet equipped with a touch-sensitive surface, a force-sensitive stylus, and an adjustable support frame. The support frame was individually adjusted to provide stable wrist or forearm support, thereby reducing fatigue during scanning. Real-time ink feedback was provided to allow participants to monitor their written or drawn output during task. Participants were instructed to write each character stroke by stroke while minimizing upper-arm and forearm movements to reduce head motion. Before the formal experiment, all participants completed in-scanner practice with the tablet by writing five example words. Each block began with a 200-ms fixation cross, followed by image presentation, with each item displayed for 1.5 s. After each image appeared, participants had 3–5 s to respond, depending on the stroke count of the target characters in the experimental condition. After each line drawing appeared, participants had 3 s to respond. Blocks were separated by fixation intervals of 9–12 s, yielding a total run duration of 11 min 46 s.

### MRI data acquisition and preprocessing

#### MRI data acquisition

MRI data were acquired using a 3.0-T Siemens Magnetom Prisma scanner at the MRI Center of the Institute of Biophysics, Chinese Academy of Sciences. High-resolution structural images were acquired using a T1-weighted magnetization-prepared rapid acquisition gradient-echo (MPRAGE) sequence (repetition time (TR) = 2200 ms; echo time (TE) = 3.49 ms; 192 sagittal slices; field of view (FOV) = 256 mm; voxel size = 1×1×1 mm).

Task and resting-state functional images were acquired using a multiband echo-planar imaging (EPI) sequence (multiband factor = 2; TR = 1500 ms; TE = 30 ms; 40 axial slices; FOV = 192 mm; voxel size = 3×3×3 mm). Task fMRI was acquired throughout the full duration of each task run, with an additional 9-s dummy period at the start of each run to allow for magnetization stabilization. Resting-state scans were acquired over 15 min in two runs of 8 min and 7 min. Diffusion-weighted images were collected using a high–angular-resolution diffusion imaging (HARDI) protocol (TR = 4000 ms; TE = 79 ms; voxel size = 1.5×1.5×1.5 mm; field of view = 192 mm). The sequence included 64 diffusion directions at *b* = 1000 s/mm^2^ and 64 directions at *b* = 2000 s/mm^2^, and five non-diffusion-weighted (*b* = 0 s/mm^2^) volumes. Structure, resting-state and diffusion scans were acquired on separate days from the task fMRI sessions to minimize fatigue.

#### Preprocessing of MRI data

Structural, task-based and resting-state fMRI data were preprocessed using *fMRIPrep* ^58,59^ followed by *XCP-D* ^60^. Structural T1-weighted images underwent intensity non-uniformity correction ^61^, skull stripping, tissue segmentation, and cortical surface reconstruction using FreeSurfer’s recon-all pipeline ^62^. fMRI data underwent susceptibility distortion correction using field maps, co-registration of the BOLD reference image to each participant’s T1-weighted anatomical image with FreeSurfer’s bbregister, head-motion correction with FSL’s mcflirt, slice-timing correction with AFNI’s 3dTshift, and cortical surface reconstruction using FreeSurfer’s recon-all pipeline. Data were then projected to grayordinates by generating HCP fsLR 64k cortical meshes with wb_command and resampling BOLD time series to the cortical surface and subcortical grey-matter structures ^51^. Finally, time series were despiked and temporally detrended using AFNI’s 3dDespike to remove transient outliers and linear or quadratic drifts ^63^. Resting-state fMRI data underwent the same preprocessing steps, followed by additional denoising procedures. These included 36-parameter confound regression, incorporating 24 motion parameters together with mean white-matter, cerebrospinal fluid and global signals, and band-pass filtering (0.01–0.08 Hz) to reduce high-frequency physiological noise and low-frequency scanner drift ^64-67^.

High-angular-resolution diffusion-weighted imaging (HARDI) data were preprocessed using QSIPrep and reconstructed for tractography using the QSIRecon MRtrix3 workflow ^68^. The T1-weighted anatomical reference image was first reoriented into AC–PC alignment using a 6-degree-of-freedom transform estimated from affine registration to the MNI152NLin2009cAsym template. Nonlinear registration to the same template was then performed using symmetric normalization in ANTs. Brain extraction was performed with SynthStrip ^69^, and tissue segmentation was obtained using SynthSeg from FreeSurfer 7.3.1^70^.

Diffusion preprocessing followed the standard QSIPrep workflow. Raw diffusion images were denoised using Marchenko–Pastur principal component analysis as implemented in MRtrix3 dwidenoise, with an automatically determined five-voxel window ^71,72^. Gibbs ringing artifacts were then removed using MRtrix3 *mrdegibbs* ^73^. Volumes with b-values below 100 s/mm^2^ were treated as b0 images. When multiple DWI sequences were acquired, the mean intensity of each diffusion series was normalized so that the mean b0 intensity was matched across sequences. Susceptibility-related and eddy-current distortions, as well as head motion, were corrected using FSL eddy ^74^. Eddy was run with outlier replacement, post-correction shell alignment, a q-space smoothing factor of 10, five iterations, and 1000 voxels for hyperparameter estimation ^75^. A quadratic first-level model and a linear second-level model were used to model eddy-current-related spatial distortions. After correction and resampling, B1 field inhomogeneity was corrected using MRtrix3 *dwibiascorrect* with the N4 algorithm ^61^. The preprocessed DWI series was resampled to AC–PC space at 1.5-mm isotropic resolution. Framewise displacement and slice-wise cross-correlation metrics were computed for quality control.

Whole-brain tractography was performed using the QSIRecon *mrtrix_multishell_msmt_ACT-hsvs* workflow. This workflow estimated multi-tissue fiber orientation distributions from multi-shell HARDI data using multi-shell multi-tissue constrained spherical deconvolution, including separate response functions for white matter, gray matter and cerebrospinal fluid ^76^. Probabilistic streamline tractography was then performed from the white-matter fiber orientation distributions using the second-order integration over fiber orientation distributions algorithm, iFOD2, in MRtrix3^77^. Anatomically constrained tractography was implemented using tissue boundaries derived from the T1-weighted segmentation and the hybrid surface–volume segmentation procedure used by the ACT-HSVS workflow ^78^. Streamline weights were subsequently estimated with SIFT2 to improve the correspondence between streamline density and the underlying fibre-density distribution ^79^. The resulting whole-brain tractograms were visually inspected before region-to-region tract extraction and structural connectivity analyses.Participants were excluded for excessive head motion if any task run exceeded 3 mm translation, 3° rotation or a mean framewise displacement greater than 0.2 mm. No participant in the CAS dataset met these exclusion criteria. Task fMRI data were spatially smoothed on the cortical surface using a 2.25-mm full-width at half-maximum kernel ^80^ to improve signal-to-noise while preserving spatial specificity.

### Peking University Dataset

#### Participants

We used a separate multimodal word-processing dataset collected at Peking University^19^ to evaluate the robustness and generalizability of our findings. The original sample comprised 101 healthy native Chinese speakers (50 women; mean age + SD, 23.0 + 2.2 years). One male participant was excluded due to excessive head motion (> 2.5 mm), yielding a final sample of 100 right-handed participants. All participants reported normal hearing, normal or corrected-to-normal vision, and no history of neurological or language disorders. Written informed consent was obtained from all participants, and the study was approved by the Institutional Review Board at Peking University.

#### Tasks

Like the CAS multimodal word-processing dataset, this dataset comprised four word-level language tasks: two comprehension tasks (listening and reading) and two production tasks (naming and writing), each with experimental and control blocks. Unlike the CAS multimodal word-processing dataset, however, the lexical items were not matched across tasks, such that each task used a distinct word set. In addition, the control contrasts and task procedures differed from those used in the corresponding CAS tasks. In the listening task, participants heard spoken Chinese nouns during the experimental blocks and judged whether each referred to a living creature; during the control blocks, they heard time-reversed speech and judged whether the speaker was male or female. In the reading task, participants viewed written Chinese nouns during the experimental blocks and judged whether each referred to a living creature, whereas during the control blocks they viewed written Chinese words and judged the number of characters in each item. In the naming task, participants viewed line drawings of common objects and named each picture aloud during the experimental blocks, whereas during the control blocks they viewed matched syllable strings and repeated them aloud. In the writing task, participants viewed object pictures and wrote the corresponding words on an MRI-compatible tablet during the experimental blocks, whereas during the control blocks they copied visually presented Chinese characters (or control symbols) with their right hand. Full details are provided in Qin et al. (2021) ^19^.

#### Data acquisition and preprocessing

MRI data were acquired on a 3T Siemens Prisma scanner (Siemens Healthineers) with a 20-channel head coil. A high-resolution T1-weighted structural image was obtained using an MPRAGE sequence (TR = 2530 ms; TE = 2.98 ms; flip angle = 7°, 1mm isotropic voxels) for anatomical reference and surface reconstruction. Task and resting-state functional images were acquired using a T2*-weighted EPI sequence with 33 contiguous axial slices covering the whole brain (TR = 2000 ms, TE = 30 ms, flip angle = 90°, matrix size = 64×64, in-plane resolution = 3.5× 3.5 mm, and slice thickness = 4.2 mm). The resting state run lasted 6 min. These data were preprocessed using the same *fMRIPrep* and *XCP-D* workflow described above.

### Human Connectome Project dataset

#### Participants

To characterize large-scale intrinsic functional gradients, we analyzed resting-state fMRI data from 245 neurologically healthy adults (130 men, 115 women) from the HCP S900 release ^51^. Participants were aged 23 to 35 years (mean + SD, 28.21 + 3.67 years). Participants were included based on HCP motion-quality metrics. Individuals were excluded if they exceeded 1.5 times the interquartile range on at least two of four summary motion measures derived from relative root mean square displacement across resting-state runs. Runs were further excluded if more than 25% of frames exceeded 0.2 mm frame-wise displacement ^81-83^. Three participants with incomplete resting-state time series were also removed. Only unrelated individuals who completed all four resting-state runs were retained for analysis.

#### MRI data acquisition and preprocessing

MRI acquisition protocols for the HCP have been described elsewhere ^51,84^. All data were collected on a customized 3-T Siemens Connectome scanner with a 100 mT/m SC72 gradient system and a 32-channel head coil. We used only the T1-weighted structural images and resting-state fMRI data in the present study.

High-resolution T1-weighted structural images were acquired using an MPRAGE sequence with 0.7-mm isotropic resolution. Resting-state fMRI data consisted of four runs, each lasting 14 min 33 s, acquired using a multiband echo-planar imaging sequence (TR = 720 ms, 2-mm isotropic) optimized for high temporal and spatial resolution.

#### Preprocessing

Resting-state fMRI data from the HCP S900 release were processed using the HCP minimal preprocessing pipelines ^51^. Structural images were corrected for spatial distortions, registered to MNI152 space using nonlinear volume-based alignment, and processed via FreeSurfer v5.3 to reconstruct cortical surfaces. Functional images underwent distortion correction, motion correction and registration to the T1-weighted image, followed by ribbon-constrained volume-to-surface mapping to generate CIFTI grayordinate data. Subcortical voxels were assigned to their corresponding structures, and cortical and subcortical data were combined in standard CIFTI grayordinate space. Surface smoothing (2-mm FWHM) was applied to reduce mixing signals across sulcal banks and areal boundaries. Resting-state fMRI data were further denoised using spatial ICA+FIX for spatially specific noise and temporal ICA ^31,85^ for removal of global structured noise removal.

### First-level activation analysis

#### CAS multimodal word-processing dataset

We estimated single-block responses for each participant, session and task for use in subsequent univariate analyses, multivariate pattern analyses, semantic decoding and gradient-based functional characterization. Single-block responses were estimated using *GLMsingle*, which improves block-wise fMRI response estimation through spatially specific hemodynamic response function selection, data-driven denoising and fractional ridge regression within a unified general linear modelling framework, thereby yielding more reliable block-wise beta estimates ^86^. For each block, onset times and durations were entered into the design matrix, with six rigid-body motion parameters included as nuisance regressors. Block-wise beta estimates were computed in CIFTI grayordinate space.

#### Peking University dataset

Because the Peking University dataset used different word items across tasks, we restricted analyses to task-wise univariate contrasts between word blocks and their matched control blocks. We estimated block-level activation maps using a standard first-level general linear model implemented in Nilearn ^87^. For each participant and task, BOLD time series were modelled with regressors for word and control blocks, convolved with a Glover hemodynamic response function. Polynomial drift terms (order 3) and six rigid-body motion parameters were included as nuisance regressors. Beta estimates were computed in CIFTI grayordinate space. We obtained word versus control statistical maps by computing t-contrast Z-maps for each task using Nilearn’s contrast estimation routines. These maps were used for univariate activation replication and gradient-based functional subregion segmentation analysis.

#### Individual-specific brain parcellation

To improve cross-participant correspondence of functional parcels, accommodate inter-individual variability in cortical organization, and increase the sensitivity of task-based analyses ^24,25,28^, all analyses were performed using individual-specific functional parcellations rather than group-level atlases or vertex-wise maps. We estimated 900 individual-specific cortical parcels for each participant using the gradient-informed multi-session hierarchical Bayesian model (gMS-HBM; ^24^) applied to the participant’s 15-min resting-state scan from the CAS dataset. Whole-brain functional connectivity gradients were first computed for each participant and used to construct individual-specific functional connectivity features in a high-dimensional gradient space. Parcellation was then initialized with a Schaefer-900 group prior pretrained on 40 HCP participants, and subject-specific parcels were estimated using gMS-HBM with w = 30, c = 30 and β = 50, following Kong et al. (2021) ^24^; see Supplementary Method for detailed information). All subsequent analyses, including activation modelling, MVPA and effective connectivity estimation, were performed at the individual-specific parcel level.

For the Peking University dataset, the 6-min resting-state scan was insufficient to support stable estimation of individual-specific parcellations. We therefore concatenated each participant’s resting-state time series with residual BOLD time series from the task runs after regressing out task effects, and used this combined intrinsic signal to compute individual-specific parcellations ^29,88-91^. Full procedural details are provided in the Supplementary Methods (*Individual-specific brain parcellation based on gMS-HBM*).

#### Group-level activation analysis and conjunction mapping

To identify regions consistently engaged across listening, reading, naming and writing, we performed parcel-wise group-level analyses on activation estimates derived from individual-specific parcellations, followed by conjunction mapping across tasks. For each participant, session and task, we averaged block-wise activation estimates within each parcel separately for the experimental and control conditions. Because the experimental condition contained more blocks than the control condition, we matched the number of experimental blocks to the number of control blocks within each session when computing condition averages, to ensure comparable signal-to-noise ratios between conditions.

For each task (listening, naming, reading and writing), we fitted a linear mixed-effects model separately for each parcel, with condition (experimental = 1, control = 0) as the fixed effect of interest, to estimate the group-level activation difference between word and control blocks. Specifically, for each participant *s*, session *k*, and parcel *i*, the activation estimate *y_i,s,k_* was modeled as:

$$y_{i,s,k}=\beta_{0,i}+\beta_{1,i}X_{s,k}+u_{s,i}+v_{k,i}+\varepsilon_{i,s,k},$$

where *β*_0,*i*_ was the parcel-specific intercept, *X_s,k_* was a binary indicator coding condition (experimental condition = 1, control condition = 0), *β*_1,*i*_ was the parcel-wise fixed effect indexing the activation difference between the experimental and control conditions, $u_{s,i}∼𝒩(0,\sigma_{u,i}^{2})$ was the participant-specific random intercept, $v_{k,i}∼𝒩(0,\sigma_{v,i}^{2})$ was the session-specific random intercept, and $\varepsilon_{i,s,k}∼𝒩(0,\sigma_{\varepsilon ,i}^{2})$ was the residual error term. We fitted the models in R 4.4.0 using the *lmerTest* package. Across the 900 parcels, *p* values were corrected for multiple comparisons using false discovery rate (FDR) correction at *q* < 0.05.

To identify regions consistently engaged across listening, reading, naming and writing, we applied the minimum-T conjunction method in SPM (https://www.fil.ion.ucl.ac.uk/spm/) to the group-level activation maps from the four word tasks. To further assess the robustness of these findings, we repeated the same mixed-effects analysis at the vertex level and generated vertex-wise activation and conjunction maps.

#### Analytical framework for identifying a sensorimotor–semantic interface

Based on the activation and conjunction analyses described above, we identified candidate regions for a sensorimotor–semantic interface. These regions were then evaluated against five a priori, location-agnostic criteria. We first tested whether they showed a comprehension–production dissociation (C1), indexed by stronger univariate responses during production than comprehension and by separable multivariate activation patterns across the two task domains. We next tested whether semantic representations were selectively enhanced during production relative to comprehension (C2), by conducting semantic category decoding and comparing decoding accuracy between the two task domains. We then tested whether effective connectivity reconfigured across tasks from sensory integration during comprehension to motor-oriented routing during production (C3), by estimating directed effective connectivity in the two task domains. We next assessed internal functional differentiation supported by white-matter connectivity (C4). Specifically, we characterized local connectivity gradients and tested whether gradient-defined subregions were functionally and structurally differentially affiliated with large-scale networks and preferentially encoded semantic versus phonological or sensorimotor information. We further tested whether local functional transitions aligned with broader macroscale cortical organization (C5), by situating subregional variation along multiple cortical hierarchy maps. This analytical workflow is summarized in Fig. 1. The specific methods used to evaluate each criterion are described below.

#### Testing C1: Dissociation between comprehension and production

To test C1, we assessed whether conjunction-defined regions distinguished word comprehension from word production using two complementary analyses: one based on univariate activation magnitude and the other on multivariate activation patterns.

#### Activation-strength analysis

For each participant and each conjunction-defined region, block-wise activation estimates were averaged separately across the two comprehension tasks (listening and reading) and the two production tasks (naming and writing). Within each region, the resulting production and comprehension estimates were compared across participants using paired-sample t-tests. This analysis tested whether word production elicited stronger responses than word comprehension. P values were corrected for multiple comparisons using false discovery rate (FDR) correction at q<0.05.

#### Multivariate classification of the comprehension–production distinction

To test whether conjunction-defined regions encoded a modality-invariant distinction between comprehension and production, we used a cross-modal multivariate pattern classification framework in which the classifier was trained on one comprehension–production task pair and tested on another pair that preserved the same distinction but involved different input or output modalities. Four train–test combinations were used: the classifier was trained to distinguish listening from naming and tested on distinguishing reading from writing, and vice versa; it was also trained to distinguish listening from writing and tested on distinguishing reading from naming, and vice versa. As a control analysis, we tested whether decoding instead reflected orthographic grouping by distinguishing tasks involving written word forms (reading and writing) from tasks without written word forms (listening and naming). Using the same framework, the classifier was trained on the listening-versus-reading contrast and tested on the naming-versus-writing contrast, or vice versa.

For each participant, we extracted block-wise beta estimates for the experimental word blocks only and averaged them across the three sessions. This yielded a 9×Nv pattern matrix for each task, where 9 denotes the 4 semantic blocks and 5 phonological blocks, and Nv denotes the number of vertices within the region. For each train–test combination, these matrices were used to construct a classification dataset comprising 18 samples (two classes × nine blocks), labelled according to the dimension being tested.

Classification was performed using an RBF kernel-based support vector machine implemented in LIBSVM 3.25 within a nested leave-one-subject-out (LOSO) framework ^92,93^. In the inner loop, the penalty parameter *C* and the RBF kernel parameter *γ* were optimized by leave-one-subject-out cross-validation on the remaining participants using predefined candidate grids. Features were demeaned and min–max normalized using the training data, and the same scaling parameters were then applied to the held-out participant.

Decoding accuracy was defined as the proportion of correctly classified samples in the test set (chance = 50%). For each participant and each region, decoding accuracies were averaged across the four comprehension–production train–test combinations to obtain the mean comprehension–production decoding accuracy, and separately across the two orthographic control combinations to obtain the mean orthographic decoding accuracy. Mean decoding accuracy for each analysis was then tested against chance using one-tailed independent T tests, with Bonferroni correction across regions (Pcorrected < 0.05). To assess whether decoding preferentially captured the comprehension–production distinction rather than orthographic grouping, comprehension–production decoding accuracy was also directly compared with orthographic decoding accuracy using paired-sample t tests. A region was considered to distinguish comprehension from production only if mean comprehension–production decoding accuracy was significantly above chance and significantly greater than orthographic decoding accuracy, whereas orthographic decoding accuracy did not exceed chance. This criterion ensured that successful decoding reflected modality-invariant task demands rather than grouping by input or output format.

#### PCA-based visualization of multivariate task representations

To visualize the representational structure underlying the multivariate pattern classification results, we projected task-specific activation patterns into a low-dimensional space using PCA. PCA was applied to the same block-wise beta estimates from the experimental word blocks used in the multivariate pattern classification analyses. For each region, block-wise activation patterns were first averaged within each task across participants, yielding four group-level task-specific activation patterns, corresponding to listening, reading, naming, and writing, in the original high-dimensional feature space. These patterns were then projected into a low-dimensional representational space to define the group-level embedding used for visualization.

To visualize individual-level representations in a common space, the same procedure was applied to subject-specific block-wise beta estimates from the experimental word blocks. For each participant and each region, block-wise activation patterns were averaged within each task to yield four task-specific activation patterns, which were then projected into a low-dimensional space. Each participant’s embedding was aligned to the group-level solution using Procrustes analysis ^94-96^. This alignment preserved the internal representational structure of task representations while placing them in a shared coordinate system. All analyses were implemented in MATLAB R2021a ^97^.

#### Testing C2: Production-selective enhancement of semantic representations

To test C2, we asked whether semantic representations were selectively enhanced during word production relative to comprehension in the candidate regions identified in C1. We performed multivariate semantic category decoding separately for each of the four word-level tasks and then compared decoding accuracy between the production tasks (naming and writing) and the comprehension tasks (listening and reading).

#### Multivariate semantic category classification

For each participant and each region, block-wise beta estimates were extracted from the experimental word blocks. Within each task, activation patterns for each semantic category were averaged across the three sessions, yielding a 4×Nv feature matrix, where the four rows correspond to the semantic categories (household appliances, places, mammals and vehicles) and Nv denoted the number of vertices within the region. The four categories yielded six pairwise comparisons. For each category pair, multivariate classification was performed to distinguish the two category-specific activation patterns, using an RBF kernel-based support vector machine implemented in LIBSVM 3.25 within a nested LOSO framework same as C1 ^92,93^. In the inner loop, the penalty parameter *C* and the RBF kernel parameter *γ* were optimized by leave-one-subject-out cross-validation on the remaining participants using predefined candidate grids. Features were demeaned and min–max normalized using the training data, and the same scaling parameters were then applied to the held-out participant.

Classification accuracy was defined as the proportion of correctly classified samples in the test set (chance = 50%). For each participant, region and task, decoding accuracies were averaged across the six category pairs to yield a task-specific semantic decoding score. These task-specific scores were then averaged across listening and reading to derive a comprehension decoding score and across naming and writing to derive a production decoding score. Within each region, production and comprehension decoding scores were compared across participants using paired-sample t-tests. To further characterize the source of any production–comprehension difference, production and comprehension decoding scores were also tested separately against chance using one-sample t-tests. P values were corrected for multiple comparisons using FDR correction at *q* < 0.05.

#### Testing C3: Task-dependent reconfiguration of effective connectivity via DCM

To test whether area 55b reconfigures its directed interactions according to the direction of language processing, we used DCM implemented in SPM12. We tested the a priori prediction that lexical production, relative to lexical comprehension, would increase the directed influence from area 55b to production-related frontal and motor-effector regions. Because this hypothesis specifically concerned the interface role of area 55b, we specified a sparse area 55b-centred DCM rather than a fully connected network.

The model included eight task-relevant regions spanning input, lexical and output components of the four word-processing tasks: auditory cortex, visual cortex, area 55b, anterior fusiform cortex, inferior frontal junction, superior frontal language area, ventral laryngeal–orofacial motor cortex and dorsal hand motor cortex. Regions were defined in each participant’s individualized Schaefer–Kong 900-parcel parcellation, and DCM time series were extracted from task-responsive voxels within each region of interest (ROI). Detailed ROI definitions and volumes of interest (VOIs) extraction procedures are provided in Supplementary Methods ROI definition for dynamic causal modelling analyses.

For each participant and task, we estimated one eight-node DCM. The endogenous connectivity matrix included self-connections for all nodes and bidirectional connections between area 55b and each of the seven other nodes; direct connections among non-55b nodes were not modelled. The lexical-processing contrast between each word task and its matched control condition was specified as a modulatory input on the bidirectional area 55b-centred connections. Driving inputs were assigned to sensory input regions according to task modality. DCMs were inverted and entered into a parametric empirical Bayes framework to estimate group-level effects and empirical-prior-updated subject-level modulatory parameters.

The parameters of interest were the posterior expectations of lexical modulatory effects on each directed area 55b-centred connection. For each participant, we averaged these parameters across production tasks and comprehension tasks and computed the production–comprehension contrast, (naming + writing)/2 − (listening + reading)/2. Edge-wise group tests identified connections whose lexical modulation differed between production and comprehension, with false discovery rate correction applied across the tested directed area 55b-centred edges.

#### Testing C4: Gradient-based functional and structural differentiation within interface regions

To test C4, we asked whether candidate interface regions showed internal functional and structural differentiation organized along a semantic-to-sensorimotor transition, consistent with their proposed role as a sensorimotor–semantic interface. Specifically, we tested whether subregions located toward the association-biased end of the local connectivity gradient were more strongly linked to language networks and semantic representation, whereas subregions located toward the sensorimotor-biased end were more strongly linked to action systems, production-related responses, and articulatory representations. We further asked whether this functional differentiation was supported by dissociable white-matter connectivity to language and action networks. To test these predictions, we first estimated local connectivity gradients within each candidate region and used the dominant gradient to define subregions. We then compared these subregions in four respects: their large-scale network affiliations, quantified from whole-cortex functional connectivity profiles; their white-matter connectivity profiles, assessed using tractography-derived connectivity to language and action-network targets; their relative responses during comprehension and production tasks, assessed using univariate analysis and multivariate pattern classification; and their representational content, assessed using semantic category decoding and articulatory-feature encoding.

#### Local functional connectivity gradient analysis

To test whether candidate interface regions contained internally differentiated subregions, we used local functional connectivity gradient analysis to partition each region on the basis of similarity in whole-cortex connectivity patterns. Of the conjunction-defined regions, only area 55b and the superior frontal language area (SFL) met the criteria in C1 and C2 and were therefore carried forward to C3. In the individualized 900-parcel atlas, however, both regions were too small to support reliable subregional analyses. We therefore mapped each region to the Glasser 360 atlas ^31^ and used the corresponding Glasser parcel for subsequent gradient analyses. Specifically, we quantified surface overlap between each candidate region and Glasser parcels and selected the parcel containing the large majority of its vertices. For area 55b, 93% of vertices overlapped with Glasser area 55b, and we therefore used the full Glasser-defined 55b in the subsequent analyses. SFL was treated in the same way. Gradient estimation was performed separately in each dataset described above. For the Peking University dataset, each participant’s 6-min resting-state scan was concatenated with 15 min of residual task-fMRI time series after regressing out task effects to increase the amount of intrinsic signal for gradient estimation.

Within each participant, we computed a whole-cortex functional connectivity profile for every vertex in the region by correlating its resting-state time series with the time series of all cortical vertices in fs_LR 32k space (59,412 vertices). This yielded a vertex-by-cortex functional connectivity matrix, which was then Fisher z-transformed. These matrices were averaged across participants to obtain a group-level functional connectivity matrix for the region. Cosine similarity was then computed between vertices within the region to generate a vertex-by-vertex affinity matrix. Diffusion embedding, as implemented in BrainSpace ^94^, was applied to this affinity matrix to derive connectivity gradients. The first gradient, which captured the dominant mode of variation in local connectivity organization, was taken as the group-level principal gradient ^94-96^.

Subject-specific gradients were then estimated from each participant’s Fisher z-transformed vertex-by-cortex functional connectivity matrix using the same procedure. Cosine similarity was used to construct an individual affinity matrix, diffusion embedding was applied to derive the individual principal gradient, and each individual gradient was aligned to the corresponding group-level principal gradient using Procrustes alignment ^94^. For each participant, the aligned principal gradient assigned a gradient value for every vertex in the region. Vertices were then subdivided into high-gradient and low-gradient subregions using a median split of these values.

#### Large-scale network affiliation of gradient-defined subregions

To determine the large-scale network affiliation of the gradient-defined subregions, we first computed whole-cortex functional connectivity maps for each subregion. For each participant, the mean BOLD time series was extracted from each subregion and correlated with the time series of all cortical vertices, followed by Fisher z transformation. These seed-to-cortex connectivity maps were then averaged across participants and thresholded using FDR correction at *q* < 0.05.

We next quantified the spatial overlap between each thresholded connectivity map and several canonical network templates, including the 17-network cortical parcellation^24,98^, a fronto-temporal language network ^99^, a semantic control network ^100^, the somato-cognitive action network (SCAN; ^101^) and the action mode network ^102^. Additional language- and action-related systems were included to assess whether the overlap patterns were specific to established language networks or extended to recently proposed action-related systems. All networks were binarized in surface space, and overlap was quantified using the Dice coefficient. For each subregion, the template with the highest Dice coefficient was taken as its dominant large-scale network affiliation.

#### Relative responses during comprehension and production tasks

To determine whether gradient-defined subregions differed in their engagement during word comprehension and production, we assessed task responses using two complementary analyses: univariate activation contrasts and multivariate pattern classification. Both analyses were performed separately within the high-gradient and low-gradient subregions of each candidate region. We predicted that sensorimotor-biased subregions would show stronger production-dominant responses and stronger discrimination between comprehension and production than association-biased subregions.

#### (1) Subregional production bias in univariate activation

For each participant, block-wise activation estimates were averaged separately across the comprehension tasks (listening and reading) and the production tasks (naming and writing) for each gradient-defined subregion. A production-bias index was then computed for each subregion as the mean activation during production minus the mean activation during comprehension.

The primary analysis tested whether production bias differed between the two subregions within each candidate region. Paired-sample t-tests were used to compare the task-bias index between the high-gradient and low-gradient subregions across participants. Follow-up one-sample t-tests were then performed separately for each subregion to determine whether its production-bias index was significantly greater than zero, indicating stronger responses during production than comprehension. P values from all between-subregion and within-subregion tests across candidate regions were corrected for multiple comparisons using FDR correction at *q* < 0.05.

#### (2) Subregional decoding of the comprehension–production distinction

We next tested whether multivariate activation patterns within each gradient-defined subregion distinguished comprehension from production. To do so, we repeated the cross-modal multivariate pattern classification analysis used in C1 separately within each subregion, using only the vertices belonging to that subregion as features.

For each participant and subregion, block-wise beta estimates from the experimental word blocks of the four word-level tasks were entered into the same cross-modal classification framework described in C1. Decoding accuracies were averaged across the four comprehension–production train–test combinations to yield the mean comprehension–production decoding accuracy for each participant and subregion. The primary analysis tested whether comprehension–production decoding accuracy differed between the two subregions within each candidate region. Paired-sample t-tests were used to compare decoding accuracy between the high-gradient and low-gradient subregions across participants. Follow-up one-sample t-tests were then performed separately for each subregion to determine whether decoding accuracy exceeded chance (0.5). P values from all between-subregion and within-subregion tests across candidate regions were corrected for multiple comparisons using FDR correction at *q* < 0.05.

#### Representational content of gradient-defined subregions

To test whether gradient-defined subregions differed in representational content along the local connectivity gradient, we quantified semantic category decoding and articulatory-feature encoding within each subregion. Specifically, we asked whether association-biased subregions showed stronger semantic category decoding, whereas sensorimotor-biased subregions showed stronger articulatory-feature encoding. To address this question, we performed subregional semantic category decoding and subregional articulatory-feature encoding, and compared the resulting indices between the high-gradient and low-gradient subregions of each candidate region.

##### (1) Subregional semantic category decoding

To assess semantic category representation, we repeated the semantic decoding analysis used in C2 within each gradient-defined subregion, restricting the feature space to the vertices belonging to that subregion. Semantic category decoding was performed using the same pairwise classification procedure and nested LOSO framework as in C2. For each participant, task and subregion, decoding accuracies were averaged across the six category pairs to yield a task-specific semantic decoding score. These scores were then averaged across the four word-level tasks to obtain a single semantic decoding index for each participant and subregion. The primary analysis tested whether this semantic decoding index differed between the high-gradient and low-gradient subregions within each candidate region. Paired-sample t-tests were used for this comparison. P values from these between-subregion tests across candidate regions were corrected for multiple comparisons using FDR correction at *q* < 0.05.

##### (2) Subregional articulatory-feature encoding

To assess articulatory-feature encoding, we tested whether the high-gradient and low-gradient subregions within each candidate region differed in how strongly their activity was explained by articulatory features during word processing. This analysis focused on the listening, naming and reading tasks; the writing task was excluded because continuous hand movements disrupted the temporal correspondence between linguistic events and BOLD responses.

For each word, articulatory features were represented in a 28-dimensional binary feature space, with each dimension corresponding to one articulatory feature used in Chinese. Feature vectors were summed across syllables to obtain a single 28-dimensional articulatory vector for each word. Two sets of predictors were then constructed from the event timings: an articulatory regressor set containing word onsets, word durations and the corresponding articulatory vectors, and a word-onset-only regressor set containing the same word onset and word duration information but no articulatory features. To align these predictors with the BOLD signal, both regressor sets were resampled to the fMRI sampling grid using Lanczos interpolation and expanded into finite impulse response (FIR) design matrices with six delays, corresponding to six successive TRs and spanning 9 s ^103-106^..

For each participant and subregion, continuous BOLD time series were extracted from block onset to block offset for the listening, naming and reading tasks, and the resulting block-level time series were concatenated across tasks to form a single response vector ^107^. This procedure ensured that successful model fits reflected articulatory information that generalized across tasks rather than task-specific variance.

Two ridge-regression encoding models were then fitted separately for each subregion. The full model included the delayed articulatory regressors together with the delayed word-onset-only regressors, whereas the control model only included the delayed word-onset-only regressors. The word-onset-only model controlled for the temporal effects of word occurrence, such that the difference between the two models reflected variance uniquely attributable to articulatory features. Model fitting used nested 10-fold cross-validation repeated 100 times ^105,106^.

Prediction performance was quantified as the cross-validated correlation between predicted and observed BOLD responses, averaged across the 100 repetitions. These correlations were then converted into an articulatory-feature encoding index,

$$G=\frac{R_{\text{full}}^{2}-R_{\text{onset}}^{2}}{R_{\text{full}}^{2}+R_{\text{onset}}^{2}},$$

which captured the relative gain in explained variance attributable to the articulatory regressors beyond the word onset-only regressors. The primary analysis tested whether this encoding index differed between the high-gradient and low-gradient subregions within each candidate region. Paired-sample t-tests were used for this comparison. P values from these between-subregion tests across candidate regions were corrected for multiple comparisons using FDR correction at *q* < 0.05.

#### White-matter connectivity of anterior and posterior area 55b

To test whether the anterior–posterior functional organization of area 55b was supported by dissociable anatomical connectivity, we compared the white-matter connection profiles of anterior and posterior 55b with two canonical large-scale networks: the language network (LAN^99^ and the somato-cognitive action network (SCAN)^46^. We predicted that anterior 55b, which showed stronger semantic representations, would be more strongly connected to LAN, whereas posterior 55b, which showed stronger production-biased and articulatory-feature responses, would be more strongly connected to SCAN.

For each participant, tracts were extracted from the whole-brain tractogram using MRtrix3 tckedit ^71^. Anterior and posterior 55b seed masks were defined from the participant-specific subdivision of area 55b and transformed into the participant’s diffusion space. LAN and SCAN target masks were derived from the corresponding network masks and mapped into the same diffusion space using individual anatomical and surface registrations. For each seed–target pair, we retained streamlines whose endpoints intersected both the 55b seed mask and the target network mask. Exclusion masks were applied to remove streamlines entering the right hemisphere or left-hemisphere cortical regions outside the corresponding seed–target pair. Tract-density maps were generated from the retained streamlines and visually inspected for quality control.

We quantified connection strength using a normalized streamline-count measure. For each seed-target tract, we counted the number of retained streamlines and normalized this value by the geometric mean of the seed and target mask sizes:

$$\text{Connectivity}=\frac{N_{\text{streamlines}}}{\sqrt{V_{\text{seed}}\times V_{\text{target}}}},$$

where *N*_streamlines_ denotes the number of retained streamlines, and *V*_seed_ and *V*_target_ denote the number of voxels in the seed and target masks, respectively. This normalization reduced the influence of inter-individual differences in ROI size on the estimated connection strength ^68^. For each participant, normalized connectivity was computed separately for four seed–target pairs: anterior 55b–LAN, anterior 55b–SCAN, posterior 55b–LAN and posterior 55b–SCAN.

We tested whether anterior and posterior 55b showed dissociable structural connectivity profiles using a 2 × 2 repeated-measures ANOVA, with seed subregion (anterior 55b, posterior 55b) and network (LAN, SCAN) as within-participant factors and normalized connectivity as the dependent variable. The key effect of interest was the seed subregion × network interaction, which tested whether the anterior–posterior difference in connection strength depended on the target network. When this interaction was significant, planned simple-effects analyses compared anterior and posterior 55b connectivity separately within LAN and SCAN. Statistical inference was restricted to participants with complete connectivity estimates across all four seed-by-network conditions. Generalized η^2^ was reported for repeated-measures ANOVA effects, and Cohen’s *d* was reported for paired simple-effects comparisons.

#### Testing C5: Embedding of local functional segregation within established cortical hierarchies

To test C5, we asked whether the local functional segregation observed in candidate interface regions was embedded within broader cortical sensory-to-association hierarchies. Specifically, we tested whether parcels spanning a local sensory-adjacent to association-adjacent axis showed consistent, monotonic changes across multiple cortical hierarchy maps, and whether these local hierarchical transitions were stronger in candidate interface regions than in other word-related and language-network control regions.

#### Parcel sampling along the local sensory-to-association axis

Regions of interest were defined at the parcel level using the Schaefer–Kong 900-parcel parcellation ^24^. For each candidate region carried forward to C5, we identified three adjacent parcels arranged along a putative sensory-to-association axis: a sensory-adjacent parcel bordering unimodal or early sensory cortex, a central parcel corresponding to the candidate region itself, and an association-adjacent parcel bordering higher-order association cortex. The specific parcel selections and anatomical criteria are detailed in the Supplementary Methods (*Parcel-triplet sampling for the local sensory-to-association axis analysis*).

To assess whether any observed local hierarchical transitions were specific to putative interface regions rather than a generic property of the language network, we also defined three control regions within a canonical language network ^99^: inferior frontal gyrus (triangular part), mid–middle temporal gyrus and posterior middle temporal gyrus. For each control region, the same sampling strategy was applied, selecting a sensory-adjacent parcel, a central parcel and an association-adjacent parcel along the local cortical axis.

#### Cortical hierarchy maps

For each ROI, we examined how values from the sensory-adjacent, central and association-adjacent parcels changed across six cortical hierarchy maps spanning evolutionary, structural, transcriptomic, metabolic, cognitive, and composite dimensions of the sensory-to-association continuum. These maps were ordered from biological constraints to integrative functional organization. First, the evolutionary hierarchy was indexed by macaque-to-human cortical expansion, which quantifies the degree of cortical surface expansion from macaque to human, with lower values in primary sensory and motor cortices and higher values in transmodal association cortex ^108,109^. Second, the structural/myeloarchitectonic hierarchy was indexed by the T1w/T2w ratio, an in vivo proxy for intracortical myelin content, with heavily myelinated primary cortices at the sensory end of the hierarchy and lightly myelinated heteromodal regions at the association end ^110^. Third, the transcriptomic hierarchy was indexed by the first principal component of cortical gene-expression profiles derived from the Allen Human Brain Atlas, capturing a dominant gradient from sensory-biased to association-biased transcriptional signatures across cortex ^111^. Fourth, the metabolic hierarchy was indexed by PET-derived aerobic glycolysis, reflecting regional variation in non-oxidative glucose metabolism, which is typically lower in primary sensorimotor cortex and higher in association cortex ^112^. Fifth, the meta-analytic cognitive hierarchy was indexed by the first principal component of NeuroSynth term–activation decodings, summarizing large-scale functional specialization from lower-order sensorimotor functions to higher-order cognitive functions ^113^. Finally, the composite sensory-to-association hierarchy integrated structural, functional, metabolic and molecular features into a single cortex-wide ordering from primary sensory and motor regions to transmodal association cortex ^109^. Together, these six maps provided complementary biological and functional indices of where each parcel lay along the broader sensory-to-association hierarchy.

### Quantification of monotonic hierarchical transitions

For each ROI and each hierarchy map, monotonic ordered differences across the three sampled parcels were quantified using the Jonckheere–Terpstra test, with the expected ordering specified as sensory-adjacent < central < association-adjacent or the reverse, depending on the coding direction of the map. The resulting Z-statistic indexed local transition strength on that hierarchy map. Significance was assessed at a stringent threshold of P < 10^−4^. This yielded, for each ROI, six hierarchy-specific indices of local transition strength, one for each cortical hierarchy map.

### Multidimensional comparison of local hierarchical transition strength

To compare the overall strength of local hierarchical transitions across ROIs, we summarized the six hierarchy-specific indices of local transition strength into a single multidimensional index and then compared this index between candidate and comparison regions using exact paired permutation tests. For each ROI, the absolute Jonckheere–Terpstra Z-statistics obtained from the six hierarchy maps were treated as the radii of a six-axis radar polygon, and polygon area was computed as

$$A=\frac{1}{2}\;\sin\;\left(\frac{2\pi }{n}\right)\sum_{i=1}^{n}r_{i}r_{i+1},$$

where *r_i_ = ∣ Z_i_ ∣*, *r*_*n*+1_ = *r*_1_, and *n* = 6. Larger values indicate stronger and more consistent local transitions across the six hierarchy dimensions.

For each pairwise comparison between a candidate region and a comparison ROI, an exact paired permutation test was performed across the six matched hierarchy maps. Under the null hypothesis of no difference in overall local hierarchical transition strength, the *∣ *Z* ∣* values for each hierarchy map were either retained or swapped between the two ROIs independently, yielding all 2^6^ = 64 possible reassignment patterns. For each reassignment, polygon areas were recomputed and the area difference Δ*A* = *A*_candidate_ − *A*_comparison_ was calculated to form the exact null distribution. One-tailed *P* values were defined as the proportion of reassignment patterns for which Δ*A*_permutation_ ≥ Δ*A*_obsolute_. Statistical significance was set at *P* < 0.05.

### Statistical analysis

Unless otherwise stated, group-level statistical analyses were performed using two-sided one-sample or paired-sample t-tests, according to the corresponding hypothesis. For paired-sample tests, outliers were identified separately for each planned contrast using a predefined criterion based on the paired-difference values. Participants whose paired-difference values fell outside the group mean ± 2.5 s.d. were excluded from that specific contrast before statistical inference. Because this criterion was applied independently to each contrast, the number of retained participants could vary across tests; the retained sample size for each paired test can be inferred from the reported degrees of freedom as N = df + 1. Multiple comparisons were controlled using FDR correction for families of related tests. Effect sizes were reported as Cohen’s *d*.

## Supplementary Material

This is a list of supplementary files associated with this preprint. Click to download.

• Wuetal.Supplementary.docx

## Figures and Tables

**Table 1.** Modality-invariant lexical regions identified across comprehension and production tasks

Manuscript region	Schaefer–Kong parcel label	Glasser atlas label	Vertices	Minimum T across contrasts
IFJ	L ContA PFCl 5/7	Area IFJ L	99	3.51
SFL	L Language PFCd 2	Superior Frontal Language Area L	53	3.01
Area 55b	L SomMotB 8 /DorsAttnB PrCv 4	Area 55b L	95	3.02
Anterior fusiform gyrus	L VisualA ExStr	Ventral Fusiform L	68	3.86
